# ABT-263 enhanced bacterial phagocytosis of macrophages in aged mouse through Beclin-1-dependent autophagy

**DOI:** 10.1186/s12877-021-02173-2

**Published:** 2021-04-01

**Authors:** Yu Zhang, Li-hua Tang, Jia Lu, Li-ming Xu, Bao-li Cheng, Jun-yu Xiong

**Affiliations:** 1 Department of Anesthesiology, The First Affiliated Hospital, College of Medicine, Zhejiang University, Hangzhou, China; 2 Department of Anesthesiology, The Second Hospital of Dalian Medical University, 467 Zhongshan Road, Dalian, ZIP: 116027 Liaoning China; 3 Research Center, The Second Hospital of Dalian Medical University, Dalian, China

**Keywords:** Sepsis, Macrophage, Phagocytosis, Autophagy, Senescence

## Abstract

**Background:**

Sepsis is a critical challenge for the older adults as the immune function is less responsive by aging. Although cell numbers seem preserved in the older adults, macrophages present age-related function decline, which including reduced chemokines, phagocytosis, and autophagy. ABT-263, an inhibitor of the anti-apoptotic protein Bcl-2, is reported had a senolytic effect which can selectively clear the senescent cells in vivo and rejuvenate the aged tissues.

**Methods:**

We treated the aged (12–16 months) and young (4–6 months) C57BL/6 mouse with ABT-263, then gave the animals cecal slurry injection to induce sepsis to observe the effect of senolytic compound ABT-263 on the survival rate of sepsis. Additionally, we isolated peritoneal macrophages from the aged mouse to investigate the cell function and molecular mechanism. 3-methyladenine (3-MA), a phosphatidylinositol 3-kinases (PI3K) inhibitor, and rapamycin, an autophagy-enhancer, were used to block or mimic the autophagy, respectively. RT-PCR and Western Blot were used to detect autophagy related gene and protein changes in sepsis. EGFP-expressing *E. coli* was used as a marker to evaluate the phagocytic ability of macrophages.

**Results:**

The results showed ABT-263 treatment improved the survival rate of sepsis in the aged mouse which related to autophagy, while blocking the autophagy can eliminate this effect. It is revealed that ABT-263 enhanced the phagocytic ability of the peritoneal macrophages by increasing the Trem-2 receptor. Additionally, ABT-263 blocked the binding of Bcl-2 to Beclin-1, thus induced Beclin-1-dependent autophagy.

**Conclusion:**

ABT-263 enhanced the macrophage function in aged mouse by increasing the Trem-2 receptors and inducing a beclin-1-dependent autophagy, consequently, protected the aged mouse from sepsis.

**Supplementary Information:**

The online version contains supplementary material available at 10.1186/s12877-021-02173-2.

## Background

Sepsis is a critical challenge for the older adults. The incidence and mortality of sepsis increased remarkably with advanced age. Although the significant pathophysiologic difference lies between the of young and aged population, the majority of sepsis studies were based on young animal model [1]. Both the innate and adaptive immune functions become less responsive by aging, also termed “immunosenescence” [2], which increases the risk of infection in the older adults. On the innate immune system, although cell numbers seem preserved in the older adults, neutrophils and macrophages present age-related functional decline, including reduced chemokines, phagocytosis, and autophagy [3].

ABT-263, an inhibitor of the anti-apoptotic protein Bcl-2, was first known because of its high efficacy against lymphoid cancer and small-cell lung carcinoma [4]. Recently, it is reported that the ABT-263 had the senolytic effect which can selectively clear the senescent cells (SCs) in vivo and rejuvenate the aged tissues [5, 6]. Therefore, it is interesting to know whether this senolytic drug has positive effect on the immune function and the acute critical infectious disease like sepsis on the older adults. In this study, we investigated the effect of ABT-263 on the aged and young mouse of sepsis in vivo and its effect on the primary peritoneal macrophages from the mouse in vitro*.* We further investigated blocking or inducing autophagy with 3-Methyladenine (3-MA) and Rapamycin (Rap), respectively, on the effect of ABT-263 treatment and the possible mechanism.

## Methods

### Animal and husbandry

- Twelve to Sixteen months old (body weight 25-35 g) and 4–6 months old (body weight 19-24 g) female C57BL/6 mice were obtained from the SPF-animal center of Dalian Medical University. The 12- to 16-month-old mice which were regarded as the aged mice were equivalent of 60- to 65-year-old people in human [7]. The 4–6 months old mice were regarded as young mice in this study. All mice were kept in the animal house with food and water ad libitum, where the temperature kept 20–24 °C, humidity 40–70%, and lighting 12 h light / 12 h dark. The animals were kept for at least 7 days before the experiment to acclimate the environment. All procedures and protocols related to the animals were approved by the Institutional Animal Care and Use Committee of the Dalian Medical University and performed in accord with the legislation.

### Main reagents and antibodies

- ABT-263 (Navitoclax), 3-MA (3-methyladenine) and Rapamycin (Sirolimus) were purchased from Selleck Chemicals (TX, USA). The primary Rabbit monoclonal antibodies of Bcl-2, Bax, Beclin-1(Becn), Atg5, LC-3, Trem-2, and Tubulin-α conjugated with Alexa Fluor® 790 were acquired from Abcam (Cambridge, UK). The secondary antibodies Goat anti-Rabbit IgG H&L (Alexa Fluor®680) was purchased from Abcam as well. The DAPI (4′, 6-diamidino-2- phenylindole) was obtained from Beyotime (Shanghai, China). The F4/80-PE anti-mouse antibody, PE Rat IgG2a isotype and biotin anti-mouse CD16/32 antibody were purchased from Biolegend (San Diego, CA). The Phallodin-iFluor 633 Conjugate was purchased from ATT Bioquest® (Sunnyvale, CA). The cell culture reagents, RPMI 1640 and FBS (fetal bovine serum) were purchased from Biological Industries (Cromwell, CT). The RT^2^ First Strand kit and Profiler PCR Array (PAXX-084) and SYBR Green qPCR master mix were purchased from Qiagen (Hilden, Germany). Proteome Profiler Array of Mouse cytokine (ARY006) was purchased from R&D systems, Inc. (MN, USA).

### Animal study design

**Fig. 1:**
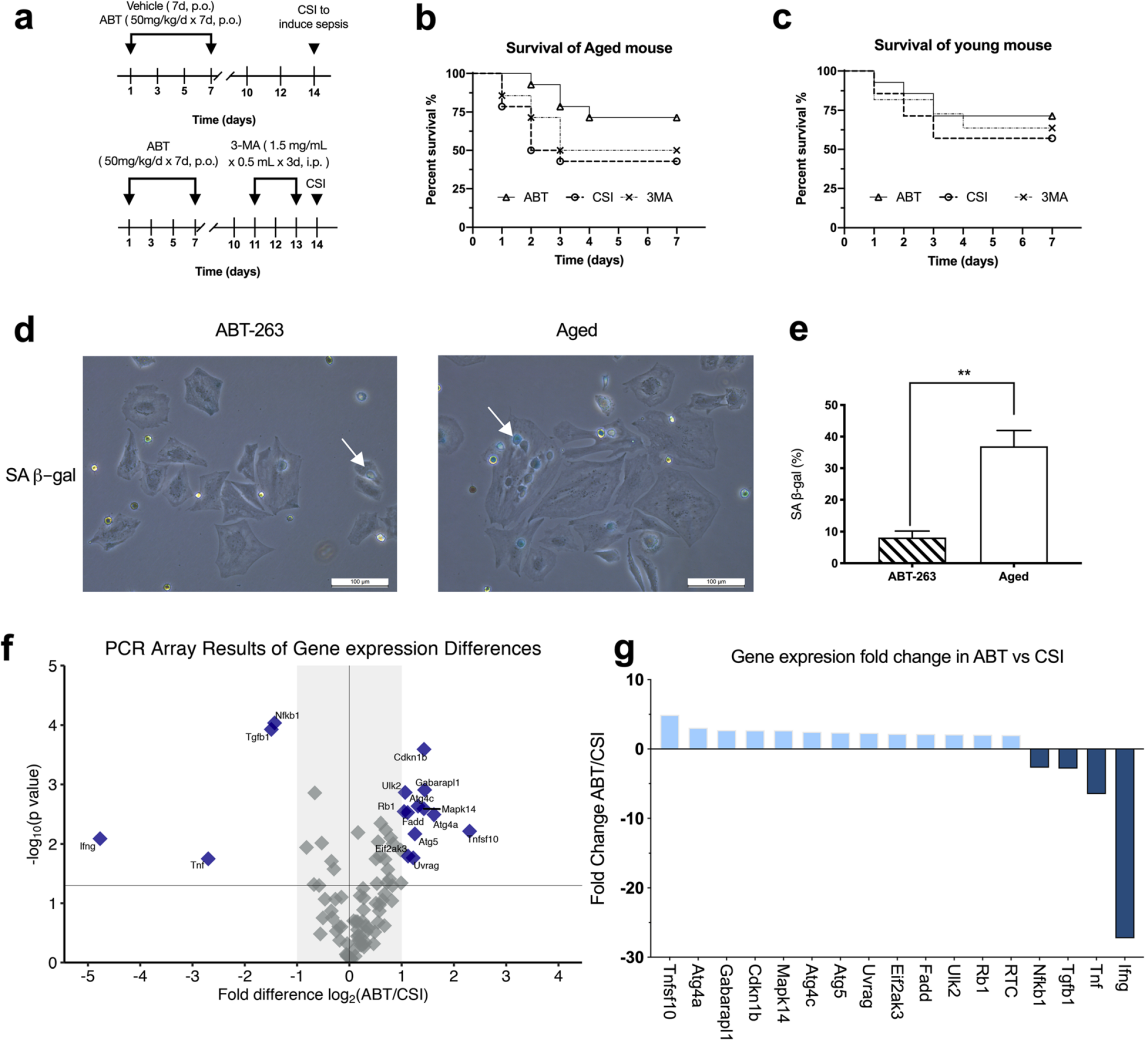
ABT-263 treatment improved the survival rate of sepsis in the aged mouse which may relate to the regulation of autophagy. **a** animal experiment design for the survival curve of sepsis. **b**-**c** The survival rates of the aged mouse which induced sepsis by cecal slurry injection (CSI) after treated by ABT-263, Vehicle and 3-MA, respectively (14 per group). The survival rate of ABT group was 71.4% (10/14), while the control group (CSI) was 42.9% (6/14), the 3-MA group was 50% (7/14). The difference between ABT and CSI group was significant (*P* = 0.038). **c** The survival rates of the young mouse induced sepsis which received the same treatments as the aged mouse. The survival rates of ABT, CSI and 3-MA were 71.4% (10/14), 57.1% (8/14) and 64.3% (9/14), respectively. How, no significant difference between those 3 groups. **d**-**e** SA- β -gal staining assay was performed in peritoneal macrophages from ABT-263 treated and Aged groups. The blue stained cells (white arrow) indicated the senescent cells and the proportions were presented in the panel **e**. ** indicated *P* < 0.05 between the two groups. **f** RNA isolated from the mouse spleen from group ABT and CSI was characterized on the Mouse RT2 Profiler PCR Array. The volcano plot of the PCR Array identified significant gene expression changes. The plot displayed statistical differences (*P* < 0.05) versus fold-change (Group ABT vs. CSI) on the y and x-axes, respectively. **g** Gene expression changes in group ABT vs. CSI. Data was plotted as relative up-regulation or down-regulation on ABT vs. CSI (*P* < 0.05). Each column is one gene and represents data from three samples for each genotype. The full gene names, their functions and *p*-value please refer to supplementary Table 1

- The animals were separated into young and aged group by age. Then, the young and aged mice were randomly divided into three subgroups: the ABT group, the 3-MA group and the CSI group (Vehicle group). We determined sample size using GraphPad Statmate (Version 2.0, GraphPad Software, Inc., La Jolla, CA). A minimal sample size of 14 in each group has a 50% power to detect an increase in survival proportion with a significance level (α = 0.05, two-tailed). Thus, fourteen mice per group were used to observe the survival rate of sepsis. In aged groups, another 4 aged mice in each subgroup were used and harvested the spleen 24 h after CSI for RT-PCR, Western blot and other analyses.
- ABT-263 was dissolved in the carrier (10% ethanol, 30% polyethylene glycol 400 and 60% Phosal 50 PG) which made a 6.5 mM solution. Each mouse in the ABT group received the oral dose of ABT-263 solution 50 mg/kg/d (approx. Volume was 160 ~ 220 μL). For the CSI group, each mouse was given the same carrier as the ABT group 200 μL orally as the vehicle control. These two groups were given the drug or vehicle once a day and continued for 7 days, then received the cecal slurry injection (CSI) to induce sepsis after a 7-day break. For the 3-MA group, the mouse was firstly given ABT-263 50 mg/kg/d the same as ABT group. Then, 3MA was dissolved in the sterile water which made a 1.5 mg/mL solution. Each mouse was injected intraperitoneally 0.5 mL/d for 3 days before received the CSI (Fig. 1 a).
- Sepsis was induced by CSI method as the literature described [8]. To be brief, prepare the cecal slurry from a fresh cecum which was dissected from a 4-month-old C57BL/6 mouse. Weigh the cecum and mix with sterile normal saline (NS) at a ratio of 0.5 ml to 100 mg of cecal content. Filter the cecal slurry (CS) twice by a sterile mesh (200-μm, Beyotime). Inject 250 μL of CS into the mouse abdomen cavity to induce acute polymicrobial abdominal sepsis.

### Mouse peritoneal macrophages isolation and primary culture

- Inject 1 mL of 3.5% Brewer thioglycolate medium into the mouse peritoneal cavity. Three days later, euthanize the mouse by cervical dislocation after rapidly inducing anesthesia by 3–5% sevoflurane. Sterilize the mouse abdomen with 75% ethanol and inject 5 mL of cold phosphate-buffered saline (PBS) into the peritoneal cavity without puncturing the bowel. Gently massage the mouse abdomen on the two sides and remove the peritoneal fluid into a centrifuge tube. Centrifuge for 10 min at 400x g at 4–8 °C. Discard the supernatant and suspend the cell pellet in RPMI 1640 medium with 10% FBS. Add 5 × 10^6^ cells into each well of a 6-well plate for the flow cytometry assay and 5 × 10^5^ cells per well into a 24-well plate for fluorescence microscopy. Culture the cells at 37 °C in a 5% CO_2_ incubator overnight. On the next day, refresh the culture medium to remove the nonadherent cells which mostly are lymphocytes. The rest of the adherent cells are mostly the macrophages, which can be easily distinguished by the morphology under a microscope.

### Cell viability assay

- Cell viability was assayed with the cell counting kit-8 (CCK-8; Dojindo, Japan) according to the manufacturer’s protocol. To be brief, the primary macrophages were planted in 96-well plates (10,000 cell/well) and incubated in RPMI 1640 with 10% FBS at 37 °C in 5% CO_2_ incubator overnight. Then, the cells were treated with several incremental concentrations of ABT-263, 3MA and rapamycin for 24 h. The next day, the cells were washed with PBS and incubated with 100 μL of CCK-8 working solution at 37 °C for 1 h. Then, read the absorbance of the wells at 450 nm using a micro-plate reader.

### SA-β-galactosidase staining

- The primary macrophages were seeded in a 6-well plate and culture at 37 °C in 5% CO_2_ incubator overnight. The SA-β-gal staining was performed using the cell senescence staining kit (Beyotime) according to the manufacturer’s instruction. After washed twice with PBS, Cells were fixed with fixation solution for 15 min at room temperature. Then, washed the cells with PBS and stained with staining solution at 37 °C overnight (Do not leave the plate in a CO_2_ incubator). Images were captured with the Leica DMI1 inverted microscope. The senescent cells were identified as blue-stained cells under microscopy. Count the SA-β-gal-positive cells in 10 randomly selected fields, and the percentages of SA-β-gal-positive cells were calculated for statistical analysis.

### Western blot assay

- The total protein of the cells or spleen tissue were extracted using 1% Triton X-100 (Beyotime) with 1x protease inhibitor cocktail (Beyotime), and then the concentration of protein was quantified using the Bradford protein assay kit (Beyotime). Twenty micrograms of protein was separated by SDS-PAGE and transferred to a PVDF membrane (0.22 μm, Bio-Rad, USA). After blocking with QuickBlock buffer (Beyotime), the membranes were incubated and shaking gently overnight at 4 °C with the primary antibody against LC-3 (1:2000), Bcl-2 (1:2000), Bax (1:2000), Beclin-1 (Becn; 1:2000), Atg5 (1:2000), Trem-2 (1:1000). All the primary and secondary antibodies were purchase from Abcam Inc.(UK). After washing with TBS-T extensively, the PVDF membranes were incubated with an appropriated Alexa Fluor® 680-conjugated secondary antibody (1:10000) for 1 h at room temperature on the next day. The bands were detected and analyzed with Odyssey CLx (LI-COR, USA) system. After the detection the PVDF membranes were stripped and incubated with Tubulin-α conjugated with Alexa Fluor® 790 (1:10000, Abcam, UK) for 2 h at room temperature, and detected the band for the loading control to normalized the interested proteins. The intensity of these bands were quantified with Image Studio software (LI-COR, version 5.2.5).

### RNA isolation, RT-PCR and PCR Array

- The total RNA of the spleen or the peritoneal macrophages were extracted and purified using the RNA-simple RNA extraction Kit (Tiangen, Beijing, China). The concentration of RNA was determined by A260/A280 using the Nanodrop 2000c (Thermo, Wilmington, DE). Equivalent amounts of RNA were reverse transcribed into cDNA using the RT^2^ First Strand kit (Qiagen) according to the kit’s protocol. The qPCR was performed using the mouse Autophagy RT^2^ Profiler PCR Array (PAXX-084; Qiagen) and SYBR Green qPCR master mix (Qiagen) according to the manufacturer’s instructions, on Light-Cycler 96 Real-Time PCR instrument (Roche, Mannheim, Germany). Gene expression data were analyzed with a web-based software from Qiagen. For the RT-PCR, primers were obtained from the web-based database Primer bank [9] and synthesized from Takara (Dalian, China). See supplementary Table 2 for the primers detail. The qPCR was also performed on Roche Light Cycler 96 using SYBR Green master mix (Tiangen). The cycle threshold (Ct) values were measured and normalized to the Ct of housekeeping gene GAPDH. The -△△Ct were calculated to indicate the relative mRNA expression of each target gene.

### Macrophage phagocytosis assay using fluorescence microscope and flow cytometer

- The phagocytosis ability of macrophages was quanified using EGFP-expressing *E. coli* as the previous literature described [10]. To be brief, add 100 μL of fresh culture medium and 10 μL of EGFP-expressing *E. coli.* Suspension (approximately 2 × 10^7^ cells) into each well of a 24-well plate which pre-cultured the peritoneal macrophages. Place the plate in a CO2 incubator for 1 h to allow the macrophages to phagocytize the bacteria. Firstly, wash once with 0.8% crystal violet water solution to quench the fluorescence of non-internalized bacteria. After washing with cold PBS, fix the cells with 4% formaldehyde at RT for 30 min. Then, wash with PBS and incubate the cells at RT for 60 min in dark with phalloidin-Alexa Fluor 633 conjugate working solution to stain the F-actin. Wash the cells with PBS and then incubate with DAPI 5 min at RT to stain the cell nuclear. Rinse once with PBS and observe using an invert fluorescence microscope (Leica DMI-3000). Count the green-fluorescence-positive cells in 10 randomly selected areas, and calculate the percentages of these cells using FIJI (ImageJ) software version 2.0 (NIH, USA).
- The flow cytometer (BD FACS Canto-II) was used to quantify the phagocytosis ability of the macrophages precisely. The primary macrophages were cultured in a 6-well plate. Add 50 μL of EGFP-expressing *E. coli.* Suspension (approximately 1 × 10^8^ cells) into the wells according to the group setting. Then place the 6-well plate in the 37 °C 5% CO2 incubator for 1 h. Wash with 0.8% CV working solution shortly to quench the fluorescence of extracellular bacteria. Wash the cells with PBS 3 times to remove any residual CV. Add 70 mM cold EDTA to detach the cells and transfer into the flow cytometry tube. Use F4/80-PE conjugated antibody (Biolegend) to mark the macrophages. Resuspend the cell pellets with 200–300 μL of PBS for flow cytometry analysis. Run each tube and acquire data for at least 10,000 events of F4/80-positive cells. BD FACS-Diva software (Version 8.0.1) was used to analyze the data and generate the contour plots.

### Cytokine antibody array assay

- Cytokine antibody array was performed with a mouse cytokine array kit (R&D Systems) according to the manufacturer’s protocol. Briefly, the mouse spleen was harvested and prepared into tissue lysate sample. After centrifuged, the membranes precoated with cytokine antibodies were incubated with supernates. After washed with washing buffer and added with detection antibody, the membranes were incubated by adding streptavidin-HRP and Chemo-Reagent Mix. The immunoblot images were captured and the intensity of each spot in the captured images was analyzed using the ChemiDoc MP system (Bio-Rad, USA).

### Statistical analysis

- The Kolmogorov-Smirnov test was performed to examine the normality of the data. The Data which passed the KS test were presented as mean ± S.D. One-way analysis of variance with Bonferroni correction, or the Student’s t test for unpaired data were performed when appropriate. Survival curves were plotted using Kaplan-Meier method and compared using the Gehan-Breslow-Wilcoxon test. GraphPad Prism (Version 8.0, GraphPad Software, Inc., La Jolla, CA) was used to perform the data analysis and generate the histograms. Statistical significance was considered when *P* < 0.05.

## Results

### ABT-263 treatment improved the survival rate of the aged sepsis mouse which may relate to the regulation of autophagy

**Fig. 2:**
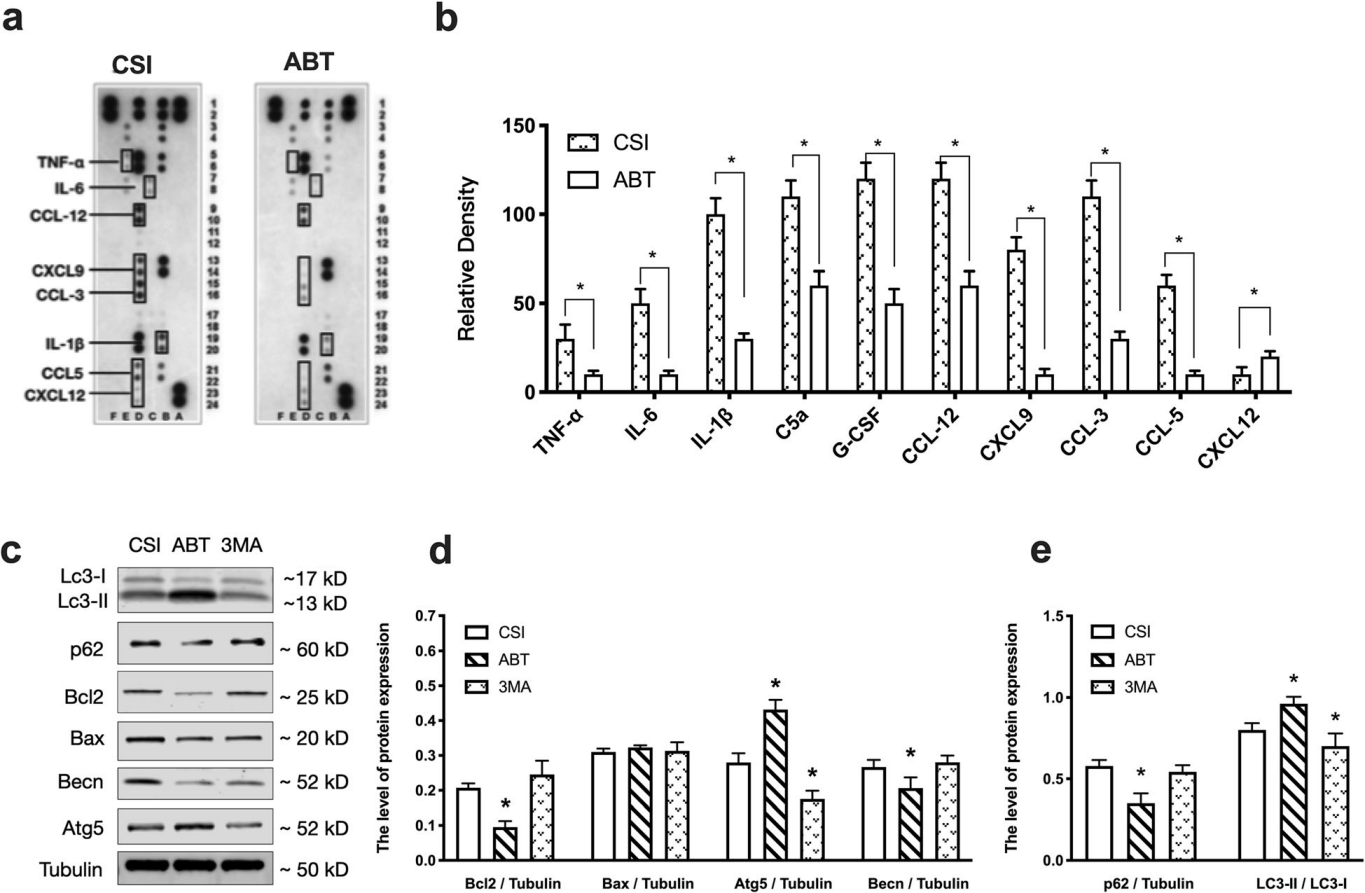
ABT-263 treatment reduced inflammatory cytokines and induced autophagy in aged mouse. **a** The chemiluminescent image of the expression of 40 different mouse cytokines with positive and negative controls in duplicates for CSI and ABT group were shown. **b** The quantitative expression of the selected cytokines found to be markedly changed in the Proteome Profiler array. **c**-**e** The representative western blotting and statistical data of proteins Lc3, p62, Bcl-2, Bax, Beclin-1, Atg5 and Tubulin-α of the mouse spleen from group CSI, ABT, and 3-MA, respectively. The data presented as the mean ± S.D. * *P* < 0.05 compared to CSI group, *n* = 4

**Fig. 3:**
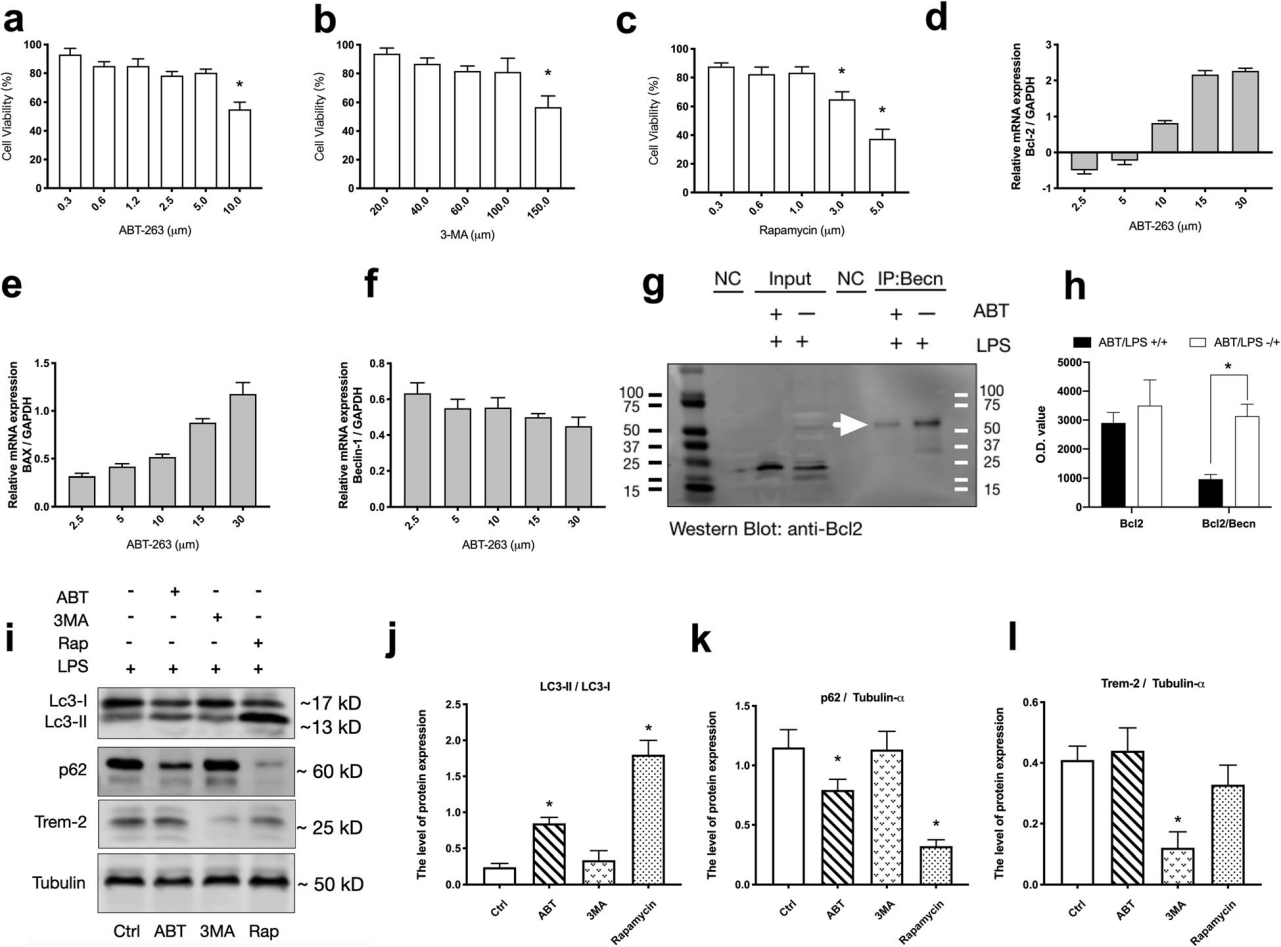
ABT-263 induced autophagy of the macrophages by blocking Bcl-2 binding to Beclin-1. **a**-**c** To avoid significant cell death becoming a confusion factor, we took the max dose at which over 80% cells were viable after 24 h as the proper concentration in the further experiments. Cell Counting Kit-8 (CCK-8) was used to quantify viable mouse peritoneal macrophages after treated by different concentrations of ABT-263, 3-MA, Rapamycin for 24 h, respectively. * indicated *P* < 0.05 compared to the first column of each figure. **d**-**f** To explore the effective dose of ABT-263 on inhibiting Bcl-2, the relative mRNA expressions of Bcl-2, BAX, Beclin-1 of the mouse peritoneal macrophages treated with five incremental concentrations of ABT-263, which were measured by real-time RT-PCR. **g**-**h** The Co-immunoprecipitation (Co-IP) of the whole cell lysates of the peritoneal macrophages. The cell lysates were firstly subjected to immunoprecipitation using Beclin-1 antibody and detected by Western blot using Bcl-2 antibody. The result showed Bcl-2/Beclin-1 complex was decreased (white arrow) when the cells were treated by ABT-263. The mouse serum was used as the Negative Control (NC). **i**-**l** The proteins, Lc3, p62, Trem-2 and Tubulin-α of the mouse peritoneal macrophages were detected by western blotting after treated by 1 μM LPS combined with 2.5 μM ABT-263, 100 μM 3-MA, and 1.0 μM Rapamycin for 24 h respectively. The data in the figures j, k, i, represented the mean ± S.D. Significant differences between groups were indicated as * *P* < 0.05 compared to the Ctrl group, *n* = 4

**Fig. 4:**
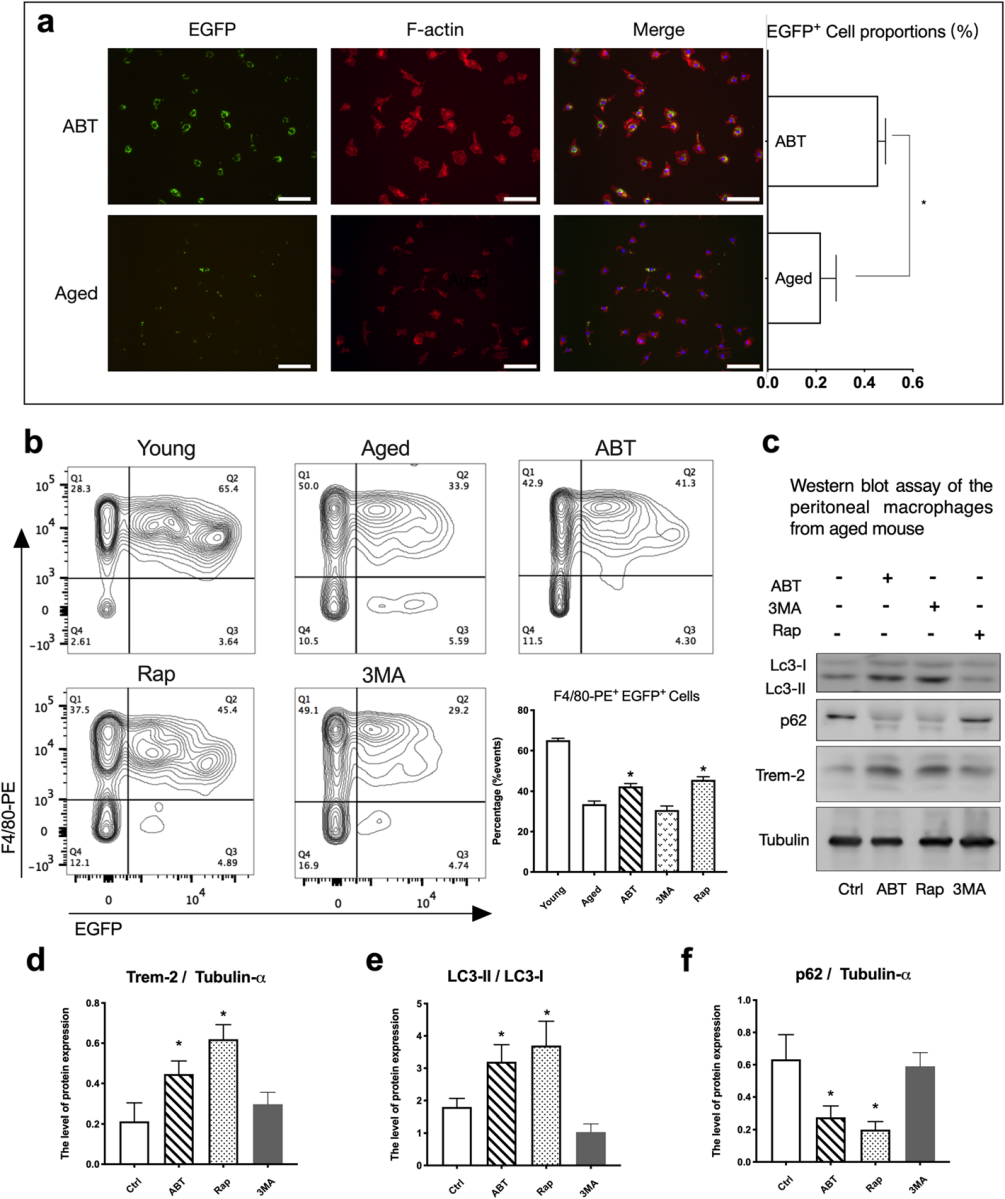
The phagocytosis of the aged mouse peritoneal macrophages was associated with the autophagy. **a** Representative multi-channel fluorescence microscope images of mouse peritoneal macrophages phagocytosed the *E. coli*. The cells were incubated with EGFP-expressing *E. coli* (green) for 1 h, followed by washing with PBS, fixation with 4% paraformaldehyde, and staining for F-actin using phalloidin-Alexa Fluor 633 conjugate working solution (red) and with DAPI (blue). The blue DAPI channel was not shown here separately. White bar = 100 μm. The proportions histogram of the EGFP positive cells counted from the fluorescence microscope images was shown on the right side. **b** Representative flow cytometry analysis of the Young, Aged, ABT, Rapamycin, and 3-MA treated groups. The peritoneal macrophages were stained with F4/80-PE after co-incubation with EGFP-expressing *E. coli*. The isotype control group was not shown in the graph. Each group repeated at least for three times. The data in this panel represented the mean ± S.D. * represented *P* < 0.05 when compared to the Aged group. **c**-**f** The proteins Lc3, p62, Trem-2 and Tubulin-α of the peritoneal macrophages from aged mouse were exmained by western blotting after treated by 2.5 μM ABT-263, 100 μM 3-MA, and 1.0 μM Rapamycin for 24 h respectively. The data in the figures d, e, f represented the mean ± S.D. Significant differences between groups were indicated as * *P* < 0.05 compared to the Ctrl group, *n* = 4

- The general condition of the aged and young mice was normal during and after drug treatment. No other abnormal condition observed in both aged and young mice before the CSI operation. When the aged mice treated with ABT-263, the survival rate of CSI-induced sepsis increased compared to the control group (10/14 vs. 6/14, *P* = 0.038). However, the survival rate of sepsis did not change significantly (7/14) when the aged mice were treated with the autophagy inhibitor 3-MA after given ABT-263 (Fig. 1 a-3). However, ABT-263 and 3-MA treatment had no effect on the survival rate of young mice (Fig. 1 a-4). As shown in Fig. 1b and c, the ratio of SA-beta-gal positive senescent peritoneal macrophages in the ABT group decreased significantly compared to the control group, which proved the senolytic effect of ABT-263 on the aged mouse as reported [5]. Because we cannot isolate the peritoneal macrophages after CSI, we use the spleen instead, as it is one of the major immunological sites for maintaining blood homeostasis [11]. To explore whether ABT-263 affected autophagy, we isolated RNA from the mouse spleen, reverse transcribed into cDNA, and used the Mouse RT^2^ Profiler PCR Array to characterize the gene expression changes. As shown in Fig. 1(f-g), when compared to the control group, the expression of the autophagy-related genes in the ABT group, such as Atg4a, Atg4c, Atg5, Ulk2, etc. were increased (*P* < 0.05), while the inflammatory genes like interferon gamma (IFNG), tumor necrosis factor (TNF), transforming growth factor (TGFB1), etc. were decreased (*P* < 0.05). The full genes name, their function and *p*-value see supplementary Table 1. In addition, we examined spleen tissue lysate using Proteome Profiler array of mouse cytokines, the result showed abt-263 treatment reduced the expression of inflammatory cytokines in aged septic mouse (Fig. 2 a-b). As shown in Fig. 2(c-e), The protein expressions in the mouse spleen were also accordant with the PCR results. The results of western blotting showed LC3-II/LC3-I, p62, Atg5 was increased in the ABT group when compared to the control group (CSI), and Bcl-2 was decreased which indicated ABT-263 induced autophagy on aged mouse.

### ABT-263 induced autophagy by blocking Bcl-2 binding to Beclin-1

- The peritoneal macrophages from the young mouse (4–6 months) were isolated to explore the mechanism of ABT-263 on macrophage phagocytosis. We used CCK-8 to quantify viable cells to determine the proper dose of ABT-263, 3-MA (an inhibitor of autophagy) and rapamycin (an inducer of autophagy). As shown in Fig. 2(a-c), the cell viability was decreased in a dose-dependent manner. To avoid significant cell death becoming a confusion factor, we took the max dose at which over 80% cells were viable after 24 h as the proper concentration. As a result, concentrations of ABT-263 2.5 μM, 3MA 100 μM and Rapamycin 1.0 μM were chosen to treat cells in the further assays.
- To explore the effective dose of ABT-263 on inhibiting Bcl-2, we examined the Bcl-2, Bax and Beclin-1 mRNA expressing on 5 incremental concentrations. The real-time PCR results (Fig. 3 d-f) showed Bcl-2 mRNA expression level was decreased at the dose of 2.5 μM ABT-263, and Bax mRNA was increased related to the incremental concentrations of ABT-263. However, Bcl-2 and Bax level were increased with the larger concentration of ABT-263, which suggested the dose of ABT-263 larger than 5 μM would cause cell apoptosis or death rather than inhibiting Bcl-2.
- Interestingly, Beclin-1 mRNA expressing seemed not affected by the dose of ABT-263. Considering ABT-263 had a high selective inhibition on Bcl-2, we futher explored the relationship Between Bcl-2 and Beclin-1. By subjecting the cell lysates of mouse peritoneal macrophages to immunoprecipitation (IP) using the Beclin-1 antibody and then detected by Western blot using the Bcl-2 antibody, the Co-IP result (Fig. 2j) showed Bcl-2-Beclin-1 complex was decreased when the cells were treated by ABT-263. The Co-IP result suggested ABT-263 prevented Bcl-2 from binding Beclin-1, which in turn, may promote Beclin-1-dependent autophagy. That also explained why Beclin-1 mRNA level not changed with incremental ABT-263 concentrations.
- After treated by the chosen proper concentrations of those drugs, the western blotting result of the peritoneal macrophages (Fig. 3 i-l) showed LC3-II/LC3-I increased in ABT and Rapamycin treated groups, while p62 decreased correspondingly in both groups. Trem-2, which served as a phagocytic receptor on macrophages, decreased markedly when treated by the autophagy inhibitor 3-MA compared to the control group. Considering the macrophages were extracted from young mouse and treated by LPS, trem-2 expression was not significantly different among control, ABT and Rapamycin groups.

### Inducing autophagy of the senescent macrophages increased Trem-2 expression and the bacterial phagocytosis

- We isolated peritoneal macrophages from both aged and young mouse to investigate the relationship between phagocytosis and autophagy. In Fig. 4, Young group indicated the peritoneal macrophages from the young mouse. ABT, Rap and 3MA indicated the peritoneal macrophages of the ages mouse treated with ABT-263 2.5 μM, rapamycin 1.0 μM, and 3-MA 100 μM for 24 h, respectively. The fluorescence microscopy images, and the statistic histogram of EGFP-positive cell proportions showed that the ABT group had a larger proportion of EGFP positive macrophages than the Aged group (Fig. 4 a). For the flowcytometry (Fig. 4 b), The F4/80-PE antibody was used to identify the peritoneal macrophages, and the EGFP-expressing *E. coli* was served as the marker of phagocytosis. The proportion of the EGFP^+^ and F4/80-PE^+^ cells in the Young group (65.17 ± 2.1%) was higher than that in the Aged group (33.63 ± 1.5%), which suggested the innate immune function of the aged mouse compromised to some extent. By inducing the autophagy with rapamycin, the EGFP^+^-F4/80^+^ cell proportion was increased (45.67 ± 1.2%), in contrast, the percentage decreased when using the autophagy inhibitor 3MA (30.73 ± 2.1%). The result of the ABT group (42.43 ± 1.4%) was similar to Rapamycin group, which suggested ABT-263 treatment induced autophagy of macrophages and increase the senescent macrophages phagocytic ability. In addition, the peritoneal macrophages collected from the aged mouse were treated by ABT-263, 3-MA and rapamycin, respectively. Examined by western blotting, the results of Lc3-I, Lc3-II, p62, Trem-2 protein expression revealed that LC3-II/LC3-I increased in ABT and Rapamycin treated groups, while p62 decreased in those groups. In contrast to the macrophages from the young mouse, however, Trem-2 increased after treated by ABT-263 and rapamycin compared to the control group (Fig. 4 c-f).

## Discussion

Senescent cells accumulated with age and contributed to the age-related diseases. As the evidences accumulated, it reveals that the impact of age on immunity is more harmful than helpful [2]. Older age is a marked risk for mortality and morbidity of sepsis largely because of the immune system dysregulation or impairment [12]. Senolytic compound was reported to target the senescent cells [13] and was suggested to increase the health and lifespan in the murine aging models [14]. However, it is unknown that the impact of senolytic compounds on the immune system on the older adults. This study showed that the senolytic compund ABT-263 treatment not only decreased the percentage of senescent macrophages, but also induced autophagy on the macrophages, which promoted phagocytosis and protected the aged mouse from sepsis.

Autophagy essentially is a process of energy recycling [15]. Inhibition of autophagy may contribute to the aging phenotype [16], while increased autophagy delays aging and extends longevity [17]. In recent years, evidences disclosed that inducing autophagy showed its protective effects against critical disease, such as heart protection during sepsis [18], ischemia/reperfusion injury protection to aged livers [19]. In this study, one of the advantages is that the Autophagy PCR Array revealed that ABT-263 activated most autophagy relate genes on the aged mouse, suggesting the autophagy plays a vital role in cell senescence and aged-related sepsis, which can help the further study on regulatory mechanisms.

Several studies have verified that ABT-263 can induced autophagy by increasing LC3-II level and suppressing p62 [20]. Although it is known that inhibition of Bcl-2 can induce autophagy [21], the mechanism are still controversial. In this study, the concentration of ABT-263 achieved in vivo was relatively low, we did not observed the significant changes on Bcl-2 protein, but only Bcl-2 mRNA changes after ABT-263 treatment. The Co-IP result showed that ABT-263 decreased the Bcl-2/Beclin-1 complex level which suggested ABT-263 may cause autophagy by dissociating Bcl-2 from Beclin-1. This result was consistent with other studies [18, 22]. However, newly evidences showed ABT-263 induced autophagy in a BAX- and BAK1-dependent manner [23]. Thus, further studies are needed to elucidate the more detailed mechanism of this compound on inducing autophagy.

To reveal the critical role of autophagy in macrophage phagocytosis, 3-MA and rapamycin were used in the study. We found that those positive effects were dismissed when inhibiting the autophagy by 3-MA, however, rapamycin had similar effects to ABT-263 on boosting phagocytosis. These findings suggested that the enhancement of phagocytosis is caused by autophagy stimulation in senescent macrophages.

Triggering receptor expressed on myeloid cells-2 (Trem-2) is a cell surface receptor mainly expressed on macrophages, which served as a phagocytic receptor. Studies showed Trem-2 contributed to enhanced bacterial clearance in vivo and protected mouse from sepsis [24]. In this study, both ABT-263 and rapamycin increased Trem-2 level of the peritoneal macrophages while 3MA was opposite. The Trem-2 levels were also consistent with the results of phagocytosis assay, which suggested that the autophagy promotes the phagocytosis by increasing the phagocytotic receptors.

Interestingly, our study appears to contradict a recent study by Li and coworkers [25]. They found 3-MA protected the mice from sepsis while rapamycin worsens the outcome. Their conclusion seems to show inhibition of autophagy might bring benefits to endotoxic shock. These opposite results could be explained by considering the major difference that the age of animal used in the study of Li were much younger (5–6 weeks) than the animal used in our study (4–6 and 12–16 months). The autophagy level declined markedly in the aged mouse compared to the young animal, which should be counted for the contradiction. The minor consideration was the difference of the method to induce sepsis. Additionally, Cecal ligation and puncture (CLP) method is more suitable for the young mouse to replicate sepsis, while the CSI method is more suitable and controllable for the aged mouse [26].

A limitation of this study is that ABT-263, as a chemotherapy drug in the first place, can induce thrombocytopenia [27], which is not suitable for the acute disease like sepsis. However, the autophagy induced by ABT-263 and rapamycin was confirmed positive effect on boosting the senescent macrophages function on aged animal model. Thus, it provided evidence for the further study on regulation of autophagy to against the immunosenescence.

## Conclusion

**Fig. 5:**
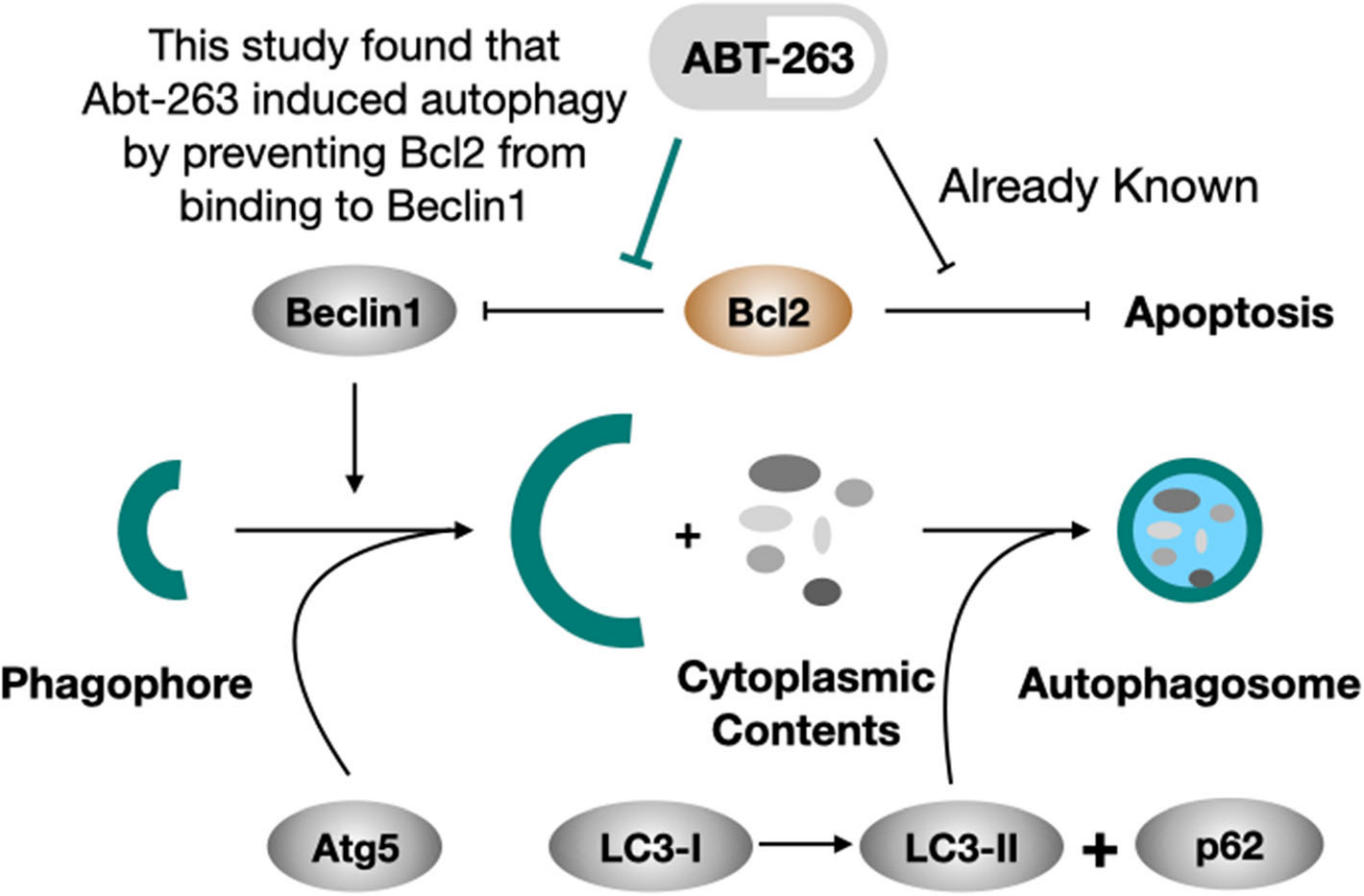
Molecular mechanisms schematic diagram. The diagram shows the possible molecular mechanism on how ABT-263 inducing Bcl-2/Beclin-1 complex dependent autophagy. This study found that ABT-263 induced Beclin1-dependent autophagy by preventing Bcl-2 from binding to Beclin-1, reducing the formation of Bcl-2/Beclin-1 complex

In conclusion, our study provides the evidence that ABT-263 enhanced the senescent macrophages function by increasing the Trem-2 receptors and inducing Beclin-1-dependent autophagy, consequently, improve the survival rate of sepsis on the aged mouse (Fig. 5). These findings suggested inducing autophagy of the aged mouse may improve the innate immune function.

## Supplementary Information

**Additional file 1 Supplementary Table S1**. PCR Array Gene Symbol Annotation and *p*-value. **Supplementary Table S2**: RT-PCR Primer list. **Supplementary Table S3**: The profiling proteins on the Mouse Cytokine Array. (PDF 222 kb)

## Data Availability

The datasets used and/or analyzed during the current study are available from the corresponding author on reasonable request.

## Abbreviations

DAPI: 4′, 6-diamidino-2- phenylindole
FBS: Fetal bovine serum
CS: Cecal slurry
CSI: Cecal slurry injection
PBS: Phosphate-buffered saline
CCK-8: Cell counting kit-8
3-MA: 3-methyladenine
Becn: Beclin-1
EGFP: Enhanced Green Fluorescent Protein
*E. coli*: *Escherichia coli*
