# Supplementary material for: ABT-263 enhanced bacterial phagocytosis of macrophages in aged mouse through Beclin-1-dependent autophagy
Source: BMC Geriatr. 2021 Apr 1;21:225. doi: 10.1186/s12877-021-02173-2 (PMC8017763; doi:10.1186/s12877-021-02173-2)

Supplementary Materials

**Table 1 PCR Array Gene Symbol Annotation and p-value**

| **Symbol** | **p-value** | **Refseq** | **Description** |
| --- | --- | --- | --- |
| Akt1 | 0.418237 | NM_009652 | Thymoma viral proto-oncogene 1 |
| Ambra1 | 0.482355 | NM_172669 | Autophagy/beclin 1 regulator 1 |
| App | 0.135151 | NM_007471 | Amyloid beta (A4) precursor protein |
| Atg10 | 0.009878 | NM_025770 | Autophagy-related 10 (yeast) |
| Atg12 | 0.056178 | NM_026217 | Autophagy-related 12 (yeast) |
| Atg16l1 | 0.016037 | NM_029846 | Autophagy-related 16-like 1 (yeast) |
| Atg16l2 | 0.050225 | NM_001111111 | Autophagy related 16 like 2 (S. cerevisiae) |
| Atg3 | 0.350418 | NM_026402 | Autophagy-related 3 (yeast) |
| Atg4a | 0.003228 | NM_174875 | Autophagy-related 4A (yeast) |
| Atg4b | 0.106587 | NM_174874 | Autophagy-related 4B (yeast) |
| Atg4c | 0.002324 | NM_175029 | Autophagy-related 4C (yeast) |
| Atg4d | 0.011438 | NM_153583 | Autophagy-related 4D (yeast) |
| Atg5 | 0.006773 | NM_053069 | Autophagy-related 5 (yeast) |
| Atg7 | 0.009177 | NM_028835 | Autophagy-related 7 (yeast) |
| Atg9a | 0.069374 | NM_001003917 | Autophagy-related 9A (yeast) |
| Atg9b | 0.019591 | NM_001002897 | ATG9 autophagy related 9 homolog B (S. cerevisiae) |
| Bad | 0.005808 | NM_007522 | BCL2-associated agonist of cell death |
| Bak1 | 0.129446 | NM_007523 | BCL2-antagonist/killer 1 |
| Bax | 0.006418 | NM_007527 | Bcl2-associated X protein |
| Bcl2 | 0.176739 | NM_009741 | B-cell leukemia/lymphoma 2 |
| Bcl2l1 | 0.007852 | NM_009743 | Bcl2-like 1 |
| Becn1 | 0.085521 | NM_019584 | Beclin 1, autophagy related |
| Bid | 0.045492 | NM_007544 | BH3 interacting domain death agonist |
| Bnip3 | 0.306074 | NM_009760 | BCL2/adenovirus E1B interacting protein 3 |
| Casp3 | 0.088415 | NM_009810 | Caspase 3 |
| Casp8 | 0.048254 | NM_009812 | Caspase 8 |
| Cdkn1b | 0.000256 | NM_009875 | Cyclin-dependent kinase inhibitor 1B |
| Cdkn2a | 0.085388 | NM_009877 | Cyclin-dependent kinase inhibitor 2A |
| Cln3 | 0.009648 | NM_009907 | Ceroid lipofuscinosis, neuronal 3, juvenile (Batten, Spielmeyer-Vogt disease) |
| Ctsb | 0.780105 | NM_007798 | Cathepsin B |
| Ctsd | 0.012641 | NM_009983 | Cathepsin D |
| Ctss | 0.791619 | NM_021281 | Cathepsin S |
| Cxcr4 | 0.175586 | NM_009911 | Chemokine (C-X-C motif) receptor 4 |
| Dapk1 | 0.250617 | NM_029653 | Death associated protein kinase 1 |
| Dram1 | 0.304528 | NM_027878 | DNA-damage regulated autophagy modulator 1 |
| Dram2 | 0.205153 | NM_026013 | VDNA-damage regulated autophagy modulator 2 |
| Eif2ak3 | 0.016035 | NM_010121 | Eukaryotic translation initiation factor 2 alpha kinase 3 |
| Eif4g1 | 0.09991 | NM_001005331 | Eukaryotic translation initiation factor 4, gamma 1 |
| Esr1 | 0.198283 | NM_007956 | Estrogen receptor 1 (alpha) |
| Fadd | 0.002981 | NM_010175 | Fas (TNFRSF6)-associated via death domain |
| Fas | 0.329499 | NM_007987 | Fas (TNF receptor superfamily member 6) |
| Gaa | 0.078918 | NM_008064 | Glucosidase, alpha, acid |
| Gabarap | 0.074422 | NM_019749 | Gamma-aminobutyric acid receptor associated protein |
| Gabarapl1 | 0.001241 | NM_020590 | Gamma-aminobutyric acid (GABA) A receptor-associated protein-like 1 |
| Gabarapl2 | 0.202991 | NM_026693 | Gamma-aminobutyric acid (GABA) A receptor-associated protein-like 2 |
| Hdac1 | 0.039005 | NM_008228 | Histone deacetylase 1 |
| Hdac6 | 0.36081 | NM_010413 | Histone deacetylase 6 |
| Hgs | 0.046587 | NM_008244 | HGF-regulated tyrosine kinase substrate |
| Hsp90aa1 | 0.434273 | NM_010480 | Heat shock protein 90, alpha (cytosolic), class A member 1 |
| Hspa8 | 0.001391 | NM_031165 | Heat shock protein 8 |
| Htt | 0.852924 | NM_010414 | Huntingtin |
| Ifng | 0.008144 | NM_008337 | Interferon gamma |
| Igf1 | 0.492931 | NM_010512 | Insulin-like growth factor 1 |
| Ins2 | 0.210306 | NM_008387 | Insulin II |
| Irgm1 | 0.7258 | NM_008326 | Immunity-related GTPase family M member 1 |
| Lamp1 | 0.511179 | NM_010684 | Lysosomal-associated membrane protein 1 |
| Map1lc3a | 0.854504 | NM_025735 | Microtubule-associated protein 1 light chain 3 alpha |
| Map1lc3b | 0.018179 | NM_026160 | Microtubule-associated protein 1 light chain 3 beta |
| Mapk14 | 0.002578 | NM_011951 | Mitogen-activated protein kinase 14 |
| Mapk8 | 0.201547 | NM_016700 | Mitogen-activated protein kinase 8 |
| Mtor | 0.045068 | NM_020009 | Mechanistic target of rapamycin (serine/threonine kinase) |
| Nfkb1 | 0.000092 | NM_008689 | Nuclear factor of kappa light polypeptide gene enhancer in B-cells 1, p105 |
| Npc1 | 0.090935 | NM_008720 | Niemann Pick type C1 |
| Pik3c3 | 0.480558 | NM_181414 | Phosphoinositide-3-kinase, class 3 |
| Pik3cg | 0.747808 | NM_020272 | Phosphoinositide-3-kinase, catalytic, gamma polypeptide |
| Pik3r4 | 0.050473 | NM_001081309 | Phosphatidylinositol 3 kinase, regulatory subunit, polypeptide 4, p150 |
| Prkaa1 | 0.026999 | NM_001013367 | Protein kinase, AMP-activated, alpha 1 catalytic subunit |
| Pten | 0.454751 | NM_008960 | Phosphatase and tensin homolog |
| Rab24 | 0.026534 | NM_009000 | RAB24, member RAS oncogene family |
| Rb1 | 0.002868 | NM_009029 | Retinoblastoma 1 |
| Rgs19 | 0.244715 | NM_026446 | Regulator of G-protein signaling 19 |
| Rps6kb1 | 0.0179 | NM_028259 | Ribosomal protein S6 kinase, polypeptide 1 |
| Snca | 0.238397 | NM_009221 | Synuclein, alpha |
| Sqstm1 | 0.134739 | NM_011018 | Sequestosome 1 |
| Tgfb1 | 0.000118 | NM_011577 | Transforming growth factor, beta 1 |
| Tgm2 | 0.613529 | NM_009373 | Transglutaminase 2, C polypeptide |
| Tmem74 | 0.547191 | NM_175502 | Transmembrane protein 74 |
| Tnf | 0.01777 | NM_013693 | Tumor necrosis factor |
| Tnfsf10 | 0.006091 | NM_009425 | Tumor necrosis factor (ligand) superfamily, member 10 |
| Trp53 | 0.081305 | NM_011640 | Transformation related protein 53 |
| Ulk1 | 0.263123 | NM_009469 | Unc-51 like kinase 1 (C. elegans) |
| Ulk2 | 0.001368 | NM_013881 | Unc-51 like kinase 2 (C. elegans) |
| Uvrag | 0.017169 | NM_178635 | UV radiation resistance associated gene |
| Wipi1 | 0.004491 | NM_145940 | WD repeat domain, phosphoinositide interacting 1 |
| Actb | 0.295082 | NM_007393 | Actin, beta |
| B2m | 0.421611 | NM_009735 | Beta-2 microglobulin |
| Gapdh | 0.242723 | NM_008084 | Glyceraldehyde-3-phosphate dehydrogenase |
| Gusb | 0.279403 | NM_010368 | Glucuronidase, beta |

Table 2 RT-PCR Primer List

| **GeneName** | **Sequence** | **Refseq** |
| --- | --- | --- |
| *Mus bec1 For* | ATGGAGGGGTCTAAGGCGTC | NM_019584 |
| *Mus bec1 Rev* | TCCTCTCCTGAGTTAGCCTCT |  |
| *mus-Bcl2-F* | GATGACTGAGTACCTGAACCG | NM_009741.5 |
| *mus-Bcl2-R* | CAGAGACAGCCAGGAGAAATC |  |
| *mus-Casp3-F* | GGGGAGCTTGGAACGCTAAG | NM_009810.3 |
| *mus-Casp3-R* | GTCCACTGACTTGCTCCCAT |  |
| *mus-Bax-F* | TGCAGAGGATGATTGCTGAC | NM_007527.3 |
| *mus-Bax-R* | CACGGAGGAAGTCCAGTGTC |  |

Table 3 The Mouse Cytokine Array coordinates

| **Coordinate** | **Target/Control** |
| --- | --- |
| A1, A2 | Reference Spot |
| A23, A24 | Reference Spot |
| B1, B2 | BLC |
| B3, B4 | C5/C5a |
| B5, B6 | G-CSF |
| B7, B8 | GM-CSF |
| B9, B10 | I-309 |
| B11, B12 | Eotaxin |
| B13, B14 | sICAM-1 |
| B15, B16 | IFN-gamma |
| B17, B18 | IL-1a |
| B19, B20 | IL-1b |
| B21, B22 | IL-1ra |
| B23, B24 | IL-2 |
| C1, C2 | IL-3 |
| C3, C4 | IL-4 |
| C5, C6 | IL-5 |
| C7, C8 | IL-6 |
| C9, C10 | IL-7 |
| C11, C12 | IL-10 |
| C13, C14 | IL-13 |
| C15, C16 | IL-12 p70 |
| C17, C18 | IL-16 |
| C19, C20 | IL-17 |
| C21, C22 | IL-23 |
| C23, C24 | IL-27 |
| D1, D2 | IP-10 |
| D3, D4 | I-TAC |
| D5, D6 | KC |
| D7, D8 | M-CSF |
| D9, D10 | JE |
| D11, D12 | MCP-5 |
| D13, D14 | MIG |
| D15, D16 | MIP-1a |
| D17, D18 | MIP-1b |
| D19, D20 | MIP-2 |
| D21, D22 | RANTES |
| D23, D24 | SDF-1 |
| E1, E2 | TARC |
| E3, E4 | TIMP-1 |
| E5, E6 | TNF-a |
| E7, E8 | TREM-1 |
| F1, F2 | Reference Spot |
| F23, F24 | PBS (Negative Control) |


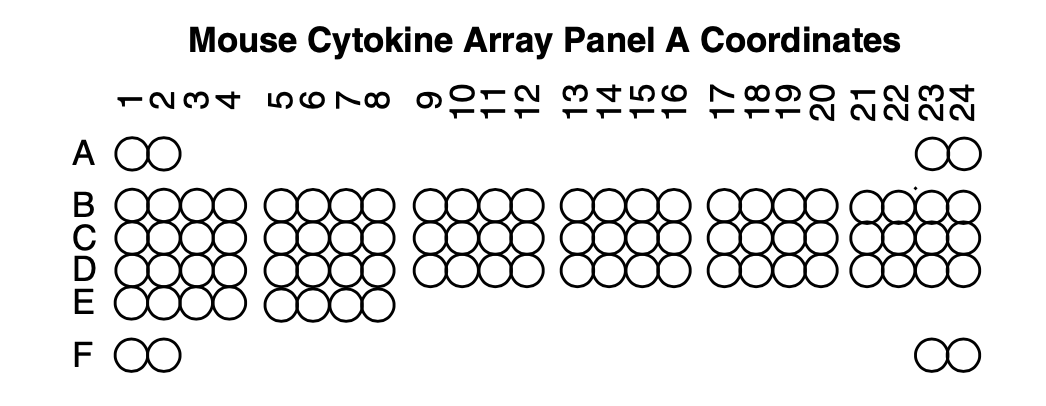

Supplement: Supplementary file 1 — Additional file 1 Supplementary Table S1. PCR Array Gene Symbol Annotation and p-value. Supplementary Table S2: RT-PCR Primer list. Supplementary Table S3: The profiling proteins on the Mouse Cytokine Array. (PDF 222 kb) [file 12877_2021_2173_MOESM1_ESM.docx]
